# DDIGIP: predicting drug-drug interactions based on Gaussian interaction profile kernels

**DOI:** 10.1186/s12859-019-3093-x

**Published:** 2019-12-24

**Authors:** Cheng Yan, Guihua Duan, Yi Pan, Fang-Xiang Wu, Jianxin Wang

**Affiliations:** 1 School of Computer Science and Engineering, Central South University, 932 South Lushan Rd, ChangSha, 410083 China; 2 School of Computer and Information,Qiannan Normal University for Nationalities, Longshan Road, DuYun, 558000 China; 3 Department of Computer Science, Georgia State University, Atlanta, GA30302 USA; 4 Biomedical Engineering and Department of Mechanical Engineering, University of Saskatchewan, Saskatoon, SKS7N5A9 Canada

**Keywords:** Drug, Drug-drug interaction, Gaussian interaction profile, RLS

## Abstract

**Background:**

A drug-drug interaction (DDI) is defined as a drug effect modified by another drug, which is very common in treating complex diseases such as cancer. Many studies have evidenced that some DDIs could be an increase or a decrease of the drug effect. However, the adverse DDIs maybe result in severe morbidity and even morality of patients, which also cause some drugs to withdraw from the market. As the multi-drug treatment becomes more and more common, identifying the potential DDIs has become the key issue in drug development and disease treatment. However, traditional biological experimental methods, including in vitro and vivo, are very time-consuming and expensive to validate new DDIs. With the development of high-throughput sequencing technology, many pharmaceutical studies and various bioinformatics data provide unprecedented opportunities to study DDIs.

**Result:**

In this study, we propose a method to predict new DDIs, namely DDIGIP, which is based on Gaussian Interaction Profile (GIP) kernel on the drug-drug interaction profiles and the Regularized Least Squares (RLS) classifier. In addition, we also use the k-nearest neighbors (KNN) to calculate the initial relational score in the presence of new drugs via the chemical, biological, phenotypic data of drugs. We compare the prediction performance of DDIGIP with other competing methods via the 5-fold cross validation, 10-cross validation and de novo drug validation.

**Conlusion:**

In 5-fold cross validation and 10-cross validation, DDRGIP method achieves the area under the ROC curve (AUC) of 0.9600 and 0.9636 which are better than state-of-the-art method (L1 Classifier ensemble method) of 0.9570 and 0.9599. Furthermore, for new drugs, the AUC value of DDIGIP in de novo drug validation reaches 0.9262 which also outperforms the other state-of-the-art method (Weighted average ensemble method) of 0.9073. Case studies and these results demonstrate that DDRGIP is an effective method to predict DDIs while being beneficial to drug development and disease treatment.

## Background

Drug-drug interactions (DDI) is defined as that a drug affects the efficacy of another drug when multi-drugs are adopted in the treatment of a disease [1]. DDIs can lead to change systemic exposure and result in variations in drug responses, which can improve the drugs efficiency and the life quality of illnesses [2]. However, DDIs also can cause serious adverse effects, drug withdrawal from the market and even the patient morality [3, 4]. Meanwhile, with the medical technology development and personalized medical requirements, more and more patients were simultaneously treated by multi-drugs and between 2009 and 2012, 38.1% of U.S. adults aging 18-44 years used three or more prescription drugs during a 30 day time period [5–7]. Therefore, identifying the potential DDIs has become a major issue in drug development and practice process.

With the high-throughput sequencing technology development, many databases related to drugs have been constructed. DrugBank database can provide drug targets, drug enzymes, drug transporters and DDIs, which are widely used in studying drug-target associations and drug repositioning [8–10]. PubChem Compound database contains the chemical substructures and their biological test results [11]. In addition, SIDER and OFFSIDES databases include drug side effects and "off-label" side effects, respectively [12, 13]. KEGG database contains drug pathways and chemical substructures [14]. TWOSIDES database contains the DDIs based on the adverse event reports in the AERS (adverse effect reactions) [13, 15].

The above mentioned databases related to drugs were extracted from the published literature and reports with experimental validation, and could provide the basis to the development of computational methods to predict new DDIs. Recently, many computational methods have been proposed to predict potential DDIs based on the assumption that similar drugs tend to interact with similar other drugs. These approaches usually used the biological network data, chemical substructure data and phenotypic data. Based on MACCS substructures of drugs, Vilar et al. developed a similarity-based model to predict new DDIs [16]. Liu et al. proposed a model to predict potential DDIs via random forest-based classification model, which also adopted a feature selection technique over the chemical substructures, protein-protein interactions between targets of drugs and target enrichment of KEGG pathways [17]. Cheng et al. proposed a method to infer novel DDIs via machine learning classifiers, whose major feature is integrating drug chemical, phenotypic and genomic properties [18]. IPFs (interaction profile fingerprints) method was proposed to predict hidden DDIs [19]. Logistic regression model was used to predict new DDIs by Takeda et al., which analyzed the effects of 2D structural similarities of drugs on DDI prediction with other pharmacokinetics (PK) and pharmacodynamics (PD) knowledge [20]. Via constructing the drug similarity based on their 2D and 3D molecular substructures, targets, side effects and known DDIs, Vilar et al. further proposed a method to predict new DDIs on a large scale data, where the key feature is capturing the characteristics of drugs by 3D substructures when 2D substructures are missing [21]. Herrero-Zazo et al. provided a computational method to predict DDIs by different types of DDIs and their mechanisms [22]. By integrating similarities from drug molecular and pharmacological phenotypes, Li et al. used a Bayesian network to provide large-scale exploration and analysis of drug combinations [23]. Through calculating the functional similarity from drug carriers, drug transporters, drug enzymes and drug targets, Ferdousi et al. developed an approach to discover new DDIs [24]. Based on the Probabilistic Soft Logic method, a computational framework was developed to discover new DDIs by integrating the multiple drug similarities and known DDIs [25]. The label propagation approach was also developed to discover new DDIs, which used drug chemical structures, side effects and off side effects [26]. In order to predict drug adverse drug reactions (ADRs), a systems pharmacology model called MEF (multiple evidence fusion) has been developed by integrating known DDIs and other similarities of drugs [27]. Based on the assumption that synergistic effects of drugs are usually similar, Network-based Laplacian regularized Least Square Synergistic (NLLSS) method was developed to predict novel DDIs [28]. Via calculating the similarities of chemical, biological, phenotypic and known DDIs of drugs, Zhang et al. proposed three ensemble methods to predict novel DDIs, which included a weight average ensemble method and two classifier ensemble methods (L1 and L2) [29].

In addition, many other approaches used quantitative structure-activity relationship (QSAR) model, clinical data and data mining to study DDIs. Matthews et al. developed 14 QSAR models to predict the cardiac adverse effects for generic pharmaceutical substances [30]. Zakharov et al. developed QSAR models to predict the likelihood of DDIs for any pair of drugs by radial basis functions with self-consistent regression (RBF-SCR) and random forest (RF) [31]. Cami et al. proposed a Predictive Pharmacointeraction Networks (PPINs) to predict novel DDIs by exploiting the known DDIs and other intrinsic and taxonomic properties of drugs and AEs [32]. Huang et al. developed a method to predict DDIs using protein-protein interaction network and clinical side effects [33]. Based on information of drug metabolism, text-mining and reasoning methods were developed to infer new DDIs [34]. Iyer et al. used the textual portion Electronic health records (EHRs) to directly discover new DDIs [35]. Banda et al. also adopted a data mining method to predict new DDIs from the EHRs [36]. Based on the k-nearest neighbor algorithm, Chen et al. proposed a model to predict DDIs which integrated nine predictors by majority voting [37]. Furthermore, the drug response prediction and drug-target interaction prediction are also the important research topics about drugs. By integrating genomic/pharmaceutical data, protein-protein interaction network, and prior knowledge of drug-target interactions with the techniques of network propagation, Wang et al. have developed a dNetFS method to prioritize genetic and gene expression features of cancer cell lines that predict drug response [38]. Based on the massively collected drug-kinase interactions and drug sensitivity datasets, Liu et al. employed a sparse linear model to infer essential kinases governing the cellular responses to drug treatments in cancer cells [39].Based on the sequence information of both targets and drugs, DeepDTA is used to predict drug-target interaction binding affinities, which is a deep-learning based model (convolutional neural networks) [40].

Although the above DDI prediction methods have achieved some good prediction results of novel DDIs and provided useful information for drug development and practice process. However, these methods did not pay enough attention to new drugs which do not have any DDIs with other drugs or cannot predict novel DDIs for new drugs because known DDIs are missing.

In this study, we develop a computational method (called DDIGIP) to predict novel DDIs based on drug Gaussian interaction profile (GIP) kernel similarity and regularized least squares (RLS) classifier. We calculate the GIP similarity of drugs by known DDIs, and then adopt the RLS method to compute the related scores of any drug pairs. In addition, when predicting DDIs for new drugs, we use the KNN method to compute the initial relational scores by similarity calculated from some important chemical, biological and phenotypic information of drugs. The drug chemical structures, drug-target interactions, drug enzymes, drug transports, drug pathways, drug indications, drug side effects and drug off side effects are all used to calculate similarity of drugs. 5-fold cross validation (5CV), 10-fold cross validation (10CV) and de novo drug validation are used to systemically assess prediction performance of DDIGIP, compared with other methods. In 5-fold cross validation, the area under the ROC curve (AUC) value of DDIGIP is 0.9600 which is slightly better than the state-of-the-art method L1 classifier ensemble (L1E) method results of 0.9570. In addition, the experimental results of 10-fold cross validation also demonstrate that DDIGIP outperforms the L1E method. In de novo drug validation, DDIGIP achieves the AUC of 0.9262, which is also better than the weighted average ensemble (WAE) method result of 0.9073. Case studies further validate the prediction ability of DDIGIP method.

## Materials

In this study, the benchmark dataset of DDIs composes of 548 drugs and 48,584 DDIs. This dataset is obtained from the TWOSIDES database. In addition, because we need to calculate the relational scores of new drugs, we also download other chemical, biological and phenotypic data from other databases to compute the similarity of drugs. The chemical data are PubChem substructures which are downloaded from the PubChem Compound database. Biological data include drug targets, drug transports, drug enzymes and drug pathways, the first three types are obtained from the DrugBank database and the last one is from the KEGG database. Furthermore, the phenotypic data composes of drug indications, drug side effects and drug off side effects. The SIDER database provided the drug indications and drug side effects, and OFFSIDES provided the drug off side effects.

Previous studies also provided the download links for these datasets [29]. Table 1 shows the relevant information which includes data type, data source and dimensionality.

**Table 1:** The description of benchmark dataset

Data type	Data	Database	dimensionality
chemical	Chemical substructures	PubChem	881
Biological	Drug-targets	DrugBank	780
	Drug transporters	DrugBank	18
	Drug enzymes	DrugBank	129
	Drug pathways	KEGG	253
Phenotypic	Drug indications	SIDER	4897
	Drug side effects	SIDER	4897
	Drug off side effects	OFFSIDES	9496
Interaction	Drug-drug interactions	TWOSIDES	Drugs:548,DDIs:48,584

## Methods

### GIP kernel similarity of drugs

The GIP kernel similarity has widely been used in other prediction issues of similar areas and achieved effective prediction performances [41–46]. RLS-Kron is provided to predict drug-target interactions based on RLS classifier of Kronecker product kernel and GIP kernel similarities of drugs and targets [41]. SDTRLS is provided to predict drug-target interactions based on integration similarity of drug GIP kernel similarity and chemical substructure similarity by the SNF method[42, 47]. LDAP is used to predict lncRNA-disease associations by using a bagging SVM classifier based on lncRNA and disease similarities which include GIP kernel similarity [43]. DNRLMF-MDA is an miRNA-disease associations prediction method based on dynamic neighborhood regularized logistic matrix factorization, which also uses the GIP kernel similarity.

We compute the GIP similarity of drugs via known DDIs in this study. We denote *D*={*d*_1_,*d*_2_,......,*d*_*N*_} as the set of *N* drugs. The known DDIs can be represented by an adjacency matrix *Y*∈*N*∗*N*. The value of *y*_*ij*_ is 1 if *d*_*i*_ and *d*_*j*_ have a known interaction, and 0 otherwise. The GIP kernel similarity between drugs *d*_*i*_ and *d*_*j*_ can be calculated as follows:

1 $$\begin{array}{@{}rcl@{}} {G_{sim}\left(d_{i},d_{j}\right)} = exp\left(-\gamma_{d} {||yd_{i}-yd_{j}||}^{2}\right) \end{array} $$

2 $$\begin{array}{@{}rcl@{}} \gamma_{d} = \gamma{^,_{d}}/\left(\frac{1}{N}\sum\limits_{i=1}^{N}{||yd_{i}||}^{2}\right) \end{array} $$

where *γ*_*d*_ is the regularization parameter of kernel bandwidth and *γd*, is set to be 1 according to previous studies [42, 44], *yd*_*i*_={*y*_*i*1_,*y*_*i*2_,......,*y*_*iN*_} is the interaction profile of drug *d*_*i*_.

### RLS classifier and prediction dDIs

The (kernel) RLS classifier is based on the assumption that similar principal (adjuvant) drugs are tended to interact with the same adjuvant (principal) drug and has been widely used in other areas [42, 48, 49]. After calculating the GIP kernel similarity *G*_*sim*_, we adopt the RLS classifier to compute the interaction probability scores of drug pairs as follows:

3 $$\begin{array}{@{}rcl@{}} \hat Y{_{p}} = G_{sim}{(G_{sim}+\sigma I)}^{-1}Y \end{array} $$

4 $$\begin{array}{@{}rcl@{}} Y_{P} = \frac{\hat Y{_{p}}+\hat Y{_{p}^{T}}}{2} \end{array} $$

where *σ* is the regularization parameter and set to be 1 according to previous study [41]. Furthermore, the *G*_*sim*_ and *I* are the GIP similarity matrix and the identity matrix, respectively. The *Y*_*p*_ is the final prediction result matrix, which is symmetric. The interacted probabilities of drug pairs are ranked in descending order. A candidate drug pair with the rank 1 is of the most possible drug pair.

### KNN for new drugs

New drugs have no any known interaction with other drugs, which makes prediction DDIs for these drugs is impossible by existing methods. Therefore, we adopt the KNN method to calculate their initial relational scores based on the integrated feature similarity of chemical structure, biological and phenotypic information.

In order to calculate the integrated feature similarity *S*_*sim*_∈*N*∗*N*, we adopt the Pearson correlation coefficient to compute similarity based on the binary vectors of drug chemical substructures, drug targets, drug transporters, drug enzymes, drug pathways, drug indications, drug side effects and drug off side effects. We can see from Table 1 that the total dimensionality of a binary vector of any drug is 21,351, whose value is 1 when the related feature is present and otherwise is 0. Specifically, the similarity of drug pair *d*_*i*_ and *d*_*j*_ is calculated as follows:

5 $$\begin{array}{@{}rcl@{}} {S{_{sim}^{i,j}}} = \frac{Cov\left(v_{d_{i}},v_{d_{j}}\right)}{{D\left(v_{d_{i}}\right)}{D\left(v_{d_{j}}\right)}} = \frac{E\left(\left(v_{d_{i}}-Ev_{d_{i}}\right)\left(v_{d_{j}}-Ev_{d_{j}}\right)\right)}{{D\left(v_{d_{i}}\right)}{D(v_{d_{j}})}} \end{array} $$

where $v_{d_{i}}$ and $v_{d_{j}}$ are the feature vectors of drugs *d*_*i*_ and *d*_*j*_, respectively. *Cov* is the covariance. *E* and *D* are the mathematical expectation and standard deviation, respectively.

After obtaining the integrated feature similarity *S*_*sim*_, we calculate the initial scores of new drugs by the KNN method. Specifically, the interaction scores *Y*_*KNN*_(*d*_*i*_,*d*_*j*_) between new drug *d*_*i*_ and another drug *d*_*j*_ can be calculated as follows:

6 $$ Y_{KNN}\left(d_{i},d_{j}\right) = \frac{\sum S{^{(i,l)}_{sim}}y_{lj}}{\sum S{^{(i,l)}_{sim}}}, d_{l} \in K_{set}  $$

where $S{^{(i,l)}_{sim}}$ is the (*i*,*l*)-th element of the integrated similarity matrix and *y*_*lj*_ is the (*l*,*j*)-th element of known DDIs matrix *Y*∈*N*∗*N*. *K*_*set*_ represents the set of top *K* nearnest neighbors according to the *S*_*sim*_ matrix. In this study, we set the value of *K* by de novo drug validation.


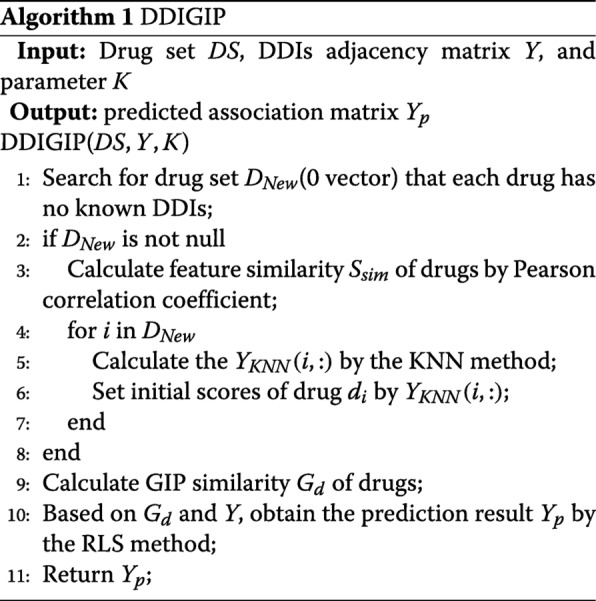


**Fig. 1:**
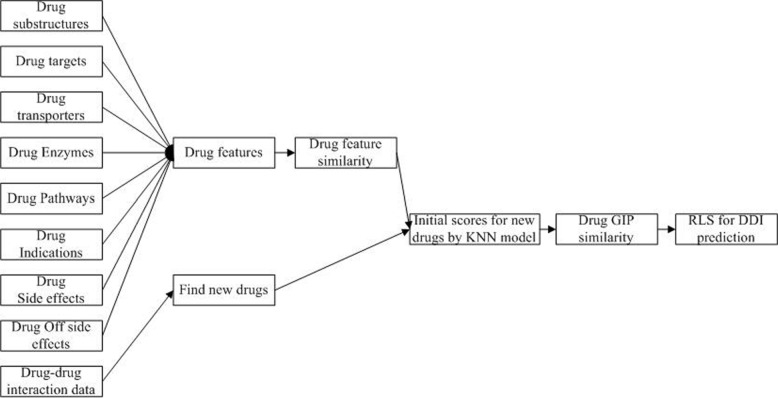
The work flow of DDIGIP

Algorithm 1 is the description of our DDIGIP method. As the 0 vectors in the DDIs adjacency matrix *Y* correspond to unknown cases, we firstly compute the initial relational interaction scores for new drugs via the KNN method which uses the feature similarity *S*_*sim*_ of drugs by integrating chemical, biological and phenotypic data. The feature similarity *S*_*sim*_ is calculated by Pearson correlation coefficient. After computing the GIP similarity *G*_*d*_ of drugs, we take the RLS classifier to calculate the interaction scores of drug pairs. The final prediction result matrix is *Y*_*p*_. Figure 1 demonstrates the work flow of DDIGIP.

## RESULTS aND dISCUSSIONS

### Benchmark evaluation and evaluation indices

5CV and 10CV are widely used to evaluate the performance for predicting drug-drug interactions [28, 29], drug-target interactions [42, 50], drug-disease interactions [51–53], lncRNA-disease associations [43, 54], miRNA-disease associations [44, 55] and so on. In this study, we evaluate the predictive performance of DDIGIP using 5CV and 10CV. In 5CV, all known DDIs are divided into 5 folds, and each fold, in turn, was left out as the test set while the rest 4 folds as the training set. In 10CV, we also divide known DDIs into 10 folds, and each fold is treated as test set in turn, while the remaining 9-folds are as the training set. We adopt 10 repeats in 5CV and 10CV. Furthermore, the actual generalization ability of predicting potential DDIs for new drugs is also an important aspect to assess the prediction performance. We thus conduct de novo drug validation to evaluate the predictive performance of DDIGIP. In de novo drug validation, we take known DDIs of each drug, in turn, and the rest DDIs of other drugs as the training set.

From a prediction method, each drug pair obtains a prediction score. Then each known interaction between two drugs in the test is ranked relative to the candidate interactions (all unknown interactions). On a specified rank threshold, TPR (true positive rate) is the fraction of known interactions that are correctly predicted, and FPR (false positive rate) is the fraction of unknown interactions that are predicted to be true interactions. The receiver operating characteristic curve (ROC) can be drawn based on various TPR and FPR values with various rank thresholds. Then we also use the area under the receiver operating curve (AUC) to measure the prediction performance of DDIGIP and other methods. The higher its AUC value is, the better prediction performance a method achieves.

### Comparison with previous methods

In this study, we compare our method with other four methods: weighted average ensemble (WAE) method, L1 classifier ensemble (L1E) method, L2 classifier ensemble (L2E) method [29] and label propagation (LP) method [26], with the same validation method in the benchmark dataset.

#### 5CV

Table 2 shows that the prediction performances of five methods in 5CV. Based on the AUC values of these methods, DDIGIP is slightly better than other methods. It shows that the GIP similarity is reasonable to use known DDIs because DDIGIP only uses known DDIs in 5CV. In addition, three integrating methods (WAE, L1E, L2E) were also achieved the good results because they integrated the neighbor recommender method, random walk method and matrix perturbation method.

**Table 2:** The prediction performances in 5CV,10CV and denovo validation, the best results are in the bold face

	The prediction performances(AUC)			
Method	Feature	5CV	10CV	Denovo
WAE	Chemical data, biological data, phenotypic data	0.9502	0.9530	0.9073
L1E	Chemical data, biological data, phenotypic data	0.9570	0.9599	*∅*
L2E	Chemical data, biological data, phenotypic data	0.9561	0.9594	*∅*
LP	Drug-sub	0.9356	0.9359	0.8993
	Drug-Label	0.9364	0.9368	0.8994
	Drug-Off Label	0.9374	0.9378	0.8997
DDIGIP	Chemical data, biological data, phenotypic data	**0.9600**	**0.9636**	**0.9262**

The *∅* represents that we did not compute the prediction performance because the prediction limit for new drugs.

#### 10CV

Table 2 also shows the prediction performances of five methods in 10CV. DDIGIP also achieved the best prediction result and its AUC value is 0.9636 which is larger than other methods WAE: 0.9530, L1E:0.9599, L2E:0.9594 and LP (max): 0.9378, respectively. By comparing the prediction performances of DDIGIP in 5CV and 10CV, DDIGIP is more effective to predict DDIs in 10CV than in 5CV. It proves that DDIGIP has better prediction ability when there are many known DDIs.

#### Denovo drug validation

In de novo drug validation, we compare DDIGIP with LP and WAE. We do not perform the de novo drug validation on other existing methods because of their prediction limit for new drugs. Similar to previous studies, we also obtain the weights of integrated methods (neighbor recommender method and random walk method) with drug chemical data, biological data and phenotypic data. Table 2 shows that DDIGIP also obtains the best prediction performance in terms of AUC (0.9262), compared with other methods (WAE: 0.9073, LP (max):0.8997). It also further indicates that the GIP similarity is effective to use known DDIs.

#### Computation time comparison

**Fig. 2:**
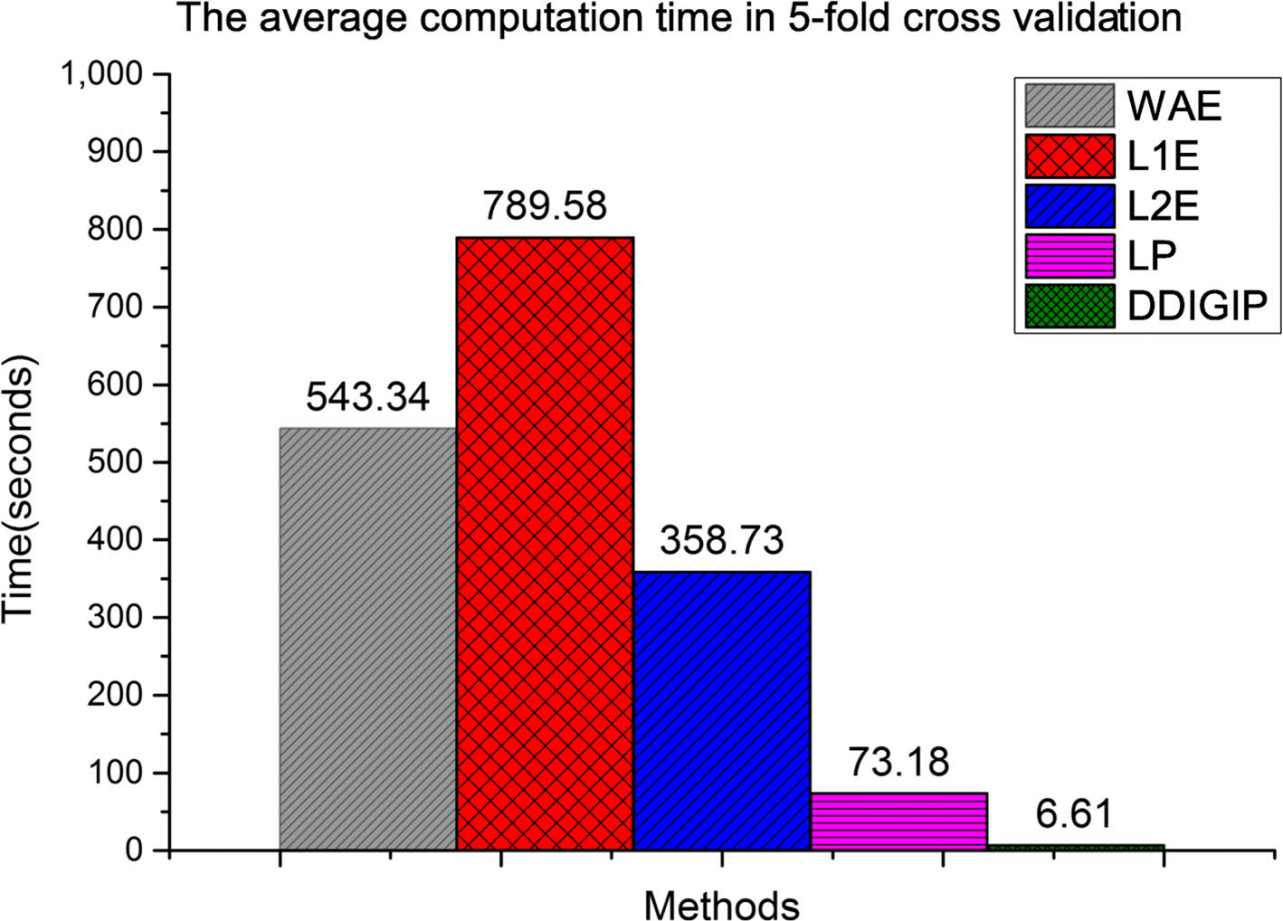
The average computation times of five methods in 5-fold cross validation

The computation time is also an important aspect to assess the performance of computational methods. In this study, we also compare the average computation time of five methods in 5CV. Figure 2 shows that the runtime of DDIGIP is less than those of other methods. In addition, since WAE, L1E and L2E are the integration method, their computation times are longer than those of LP and DDIGIP. We can see from Fig. 2 that DDIGIP runs the fastest and its computation time is 6.61 seconds in 5CV.

### Parameter analysis for *K*

**Fig. 3:**
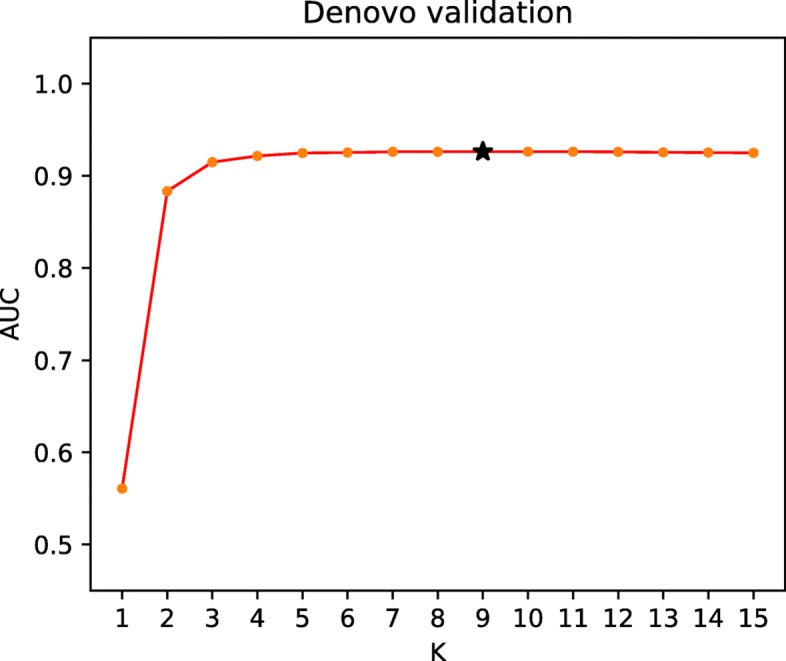
The AUC of DDIGIP under different settings of *K* in de novo drug validation, the sign ^∗^ represents the default value

In order to verify the robustness of DDIGIP, we analyze the parameters *K* that is the number of the nearest neighbors in de novo drug validation. The optimal parameter value of *K* is selected by the grid search. Figure 3 shows the AUC values of DDIGIP under variation of K ranging from 1 to 15 in de novo validation. We can see from Fig. 3 that the prediction performance has the ascending trend when K ranges from 1 to 7, while has the descending trend when K ranges from 11 to 15. In addition, DDIGIP has a relatively stable prediction performance and achieves the best prediction result (AUC:0.9262) when K is 9. It indicates that a reasonable value of K can improve the prediction performance of DDIGIP.

### Case studies

To illustrate the prediction performance of DDIGIP method, we conduct two types of case studies. The one includes the top 20 predicted DDIs under all known DDIs, in which the benchmark dataset is obtained from the TWOSIDES database while the confirmed database is DrugBanK database. Another includes top 20 the new DDIs in de novo validation of drug Ranolazine (DB00243) whose confirmed database composes of TWOSIDES database and DrugBanK database.

We can see from Table 3 that 9 out of top 20 DDIs predicted by DDIGIP are validated in DrugBank. The verification success rate is 45%. Zafirlukast (DB00549) is an oral leukotriene receptor antagonist (LTRA) drug usually used in the maintenance treatment of asthma, its metabolism can be decreased by Rabeprazole (DB01129) [56, 57]. Atazanavir (DB01072) is an antiretroviral drug of the protease inhibitor (PI) class, which is used to treat infection of human immunodeficiency virus (HIV) and its metabolism can be decreased when combining with Amlodipine (DB00381) [8, 58]. In addition, Pantoprazole (DB00213) also decreases the metabolism of Methadone (DB00333)[59]. The risk or severity of adverse effects can be increased when Atenolol (DB00335) is combined with Nadolol (DB01203), Clotrimazole (DB00257) is combined with Pregabalin (DB00230) or Enalapril (DB00584) is combined with Perindopril (DB00790) [9, 10, 60, 61]. The hypotensive activities of Nadolol (DB01203) can be increased by Propranolol (DB00571) [62]. The absorption of Cefpodoxime (DB01416) can be decreased when combining with Ranitidine (DB00863) [63]. Acebutolol (DB01193) also increases the serum concentration of Metoprolol (DB00264) [64].

**Table 3:** Top 20 new DDIs predicted by DDIGIP method

Rank	Drug ID1	Drug ID2	Evidence
1	DB00448	DB01059	Unknown
2	DB00549	DB01129	DrugBank
3	DB00991	DB00231	Unknown
4	DB00470	DB00331	Unknown
5	DB00630	DB00346	Unknown
6	DB00863	DB01416	DrugBank
7	DB01203	DB00335	DrugBank
8	DB00813	DB00535	Unknown
9	DB00257	DB00230	DrugBank
10	DB00806	DB01036	Unknown
11	DB00927	DB01193	Unknown
12	DB00333	DB00213	DrugBank
13	DB00987	DB00758	Unknown
14	DB01595	DB01137	Unknown
15	DB06151	DB01068	Unknown
16	DB00328	DB00218	Unknown
17	DB00264	DB01193	DrugBank
18	DB01072	DB00381	DrugBank
19	DB01203	DB00571	DrugBank
20	DB00584	DB00790	DrugBank

Ranolazine is an antianginal medication used in the treatment of chronic angina [10]. Table 4 shows that top 20 predicted DDIs of Ranolazine are validated in TWOSIDES database or DrugBanK database. In addition, 12 out of top 20 DDIs are simultaneously confirmed by TWOSIDES database and DrugBanK database, while the rest are confirmed by one of them. For example, the metabolism of Levothyroxine (DB00451) and Zolpidem (DB00425) can be decreased when combining with Ranolazine [15, 56]. Clopidogrel is an antiplatelet agent structurally and pharmacologically similar to ticlopidine, which is used to inhibit blood clots in a variety of conditions such as peripheral vascular disease, coronary artery disease, and cerebrovascular disease [8]. The serum concentration of Clopidogrel (DB00758) can be increased when combining with Ranolazine [15]. Similarly, the serum concentration of Simvastatin (DB00641), Acetylsalicylic (DB00945) or Metformin (DB00331) also can be increased when combining with Ranolazine [56, 65]. In addition, when Ranolazine is combined with Omeprazole (DB00338) or Acetaminophen (DB00316), its serum concentration also can be increased [15, 66].

**Table 4:** The validation result of top 20 new DDIs of drug Ranolazine (DB00243) predicted by DDIGIP method in de novo validation

Rank	Drug ID1	Drug ID2	Evidence
1	DB00243	DB00451	TWOSIDES,DrugBanK
2		DB00338	TWOSIDES,DrugBanK
3		DB00641	TWOSIDES,DrugBanK
4		DB00945	TWOSIDES,DrugBanK
5		DB00758	TWOSIDES,DrugBanK
6		DB00316	DrugBanK
7		DB00264	TWOSIDES
8		DB00695	TWOSIDES
9		DB00722	TWOSIDES
10		DB00390	TWOSIDES,DrugBanK
11		DB00448	TWOSIDES,DrugBanK
12		DB00999	TWOSIDES
13		DB00863	TWOSIDES,DrugBanK
14		DB00630	TWOSIDES
15		DB00635	DrugBanK
16		DB00213	TWOSIDES,DrugBanK
17		DB00678	TWOSIDES,DrugBanK
18		DB00425	TWOSIDES,DrugBanK
19		DB00177	TWOSIDES
20		DB00331	TWOSIDES,DrugBanK

## Conclusion

In this study, we have proposed a computational method, called DDIGIP, for DDIs prediction. The GIP similarity of drugs is calculated by the known DDIs, which makes full use of known DDIs. To our knowledge, in the previous studies the RLS-Kron method is used to predict interaction of bipartite networks, such as drug-target interaction networks, drug-disease interaction network and so on. Experiments are conducted using two different types of cross validations: 5-fold cross validation and 10-fold cross validation. The prediction ability of DDIGIP has been illustrated by comparing it with four other competing state-of-the-art methods.

Furthermore, based on Pearson correlation coefficient, we obtain a comprehensive feature similarity of drugs by integrating the chemical, biological and phenotypic data into a high dimension binary vector. In order to more effectively predict DDIs for new drugs, we also conduct de novo drug validation. We add a preprocessing step, KNN, to compute the initial relational scores according to the feature similarity of drugs. Because the vector 0 in the matrix corresponding to unknown cases or missing values rather than confirmed non-interactions, the preprocessing can improve the prediction performance.

Despite the advantages of DDIGIP as discussed above, it still has some limitations. The more effective method should be developed to integrate known DDIs with other chemical, biological and phenotypic data. In addition, other new prediction methods such as matrix completion [67], deep learning [68] and interpretable boosting model [69] could be considered. Finally, in this study, the benchmark dataset of DDIs only includes the positive samples and is an imbalanced dataset, we will also consider some other methods (SVM [70],LibD3C [71],extreme learning machine [72] and collaborative metric learning [73]) to predict DDIs when we obtain reliable negative samples in the future. We expect to develop a more effective method to predict DDIs by overcoming these limitations in the future.

## Abbreviations

10CV: 10-fold cross validation
5CV: 5-fold cross validation
AUC: Area under the receiver operating curve
DDI: Drug-drug interaction
GIP: Gaussian interaction profile
KNN: K-nearest neighbors
L1E: L1 Classifier ensemble method
L2E: L2 Classifier ensemble method
LP: Label propagation
RLS: Regularized least squares classifier
WAE: Weighted average ensemble method

## References

[1] Crowther NR, Holbrook AM, Kenwright R, Kenwright M (1997). Drug interactions among commonly used medications. chart simplifies data from critical literature review. Can Fam Phys.

[2] Venkatakrishnan K, von Moltke LL, Obach R, Greenblatt DJ (2003). Drug metabolism and drug interactions: application and clinical value of in vitro models. Curr Drug Metabolism.

[3] Quinn D, Day R (1995). Drug interactions of clinical importance. Drug Safety.

[4] Onakpoya IJ, Heneghan CJ, Aronson JK (2016). Post-marketing withdrawal of anti-obesity medicinal products because of adverse drug reactions: a systematic review. BMC Med.

[5] CDC. Health, United States, 2014 (5/2015)-hus14.pdf. http://www.cdc.gov/nchs/data/hus/hus14.pdf. Accessed 15 Nov 2017.

[6] Nahta R, Hung M-C, Esteva FJ (2004). The her-2-targeting antibodies trastuzumab and pertuzumab synergistically inhibit the survival of breast cancer cells. Cancer Res.

[7] Chou T-C (2010). Drug combination studies and their synergy quantification using the chou-talalay method. Cancer Res.

[8] Wishart DS, Knox C, Guo AC, Shrivastava S, Hassanali M, Stothard P, Chang Z, Woolsey J (2006). Drugbank: a comprehensive resource for in silico drug discovery and exploration. Nucleic Acids Res.

[9] Knox C, Law V, Jewison T, Liu P, Ly S, Frolkis A, Pon A, Banco K, Mak C, Neveu V (2010). Drugbank 3.0: a comprehensive resource for ’omics’ research on drugs. Nucleic Acids Res.

[10] Law V, Knox C, Djoumbou Y, Jewison T, Guo AC, Liu Y, Maciejewski A, Arndt D, Wilson M, Neveu V (2013). Drugbank 4.0: shedding new light on drug metabolism. Nucleic Acids Res.

[11] Wang Y, Xiao J, Suzek TO, Zhang J, Wang J, Bryant SH (2009). Pubchem: a public information system for analyzing bioactivities of small molecules. Nucleic Acids Res.

[12] Kuhn M, Campillos M, Letunic I, Jensen LJ, Bork P (2010). A side effect resource to capture phenotypic effects of drugs. Mole Syst Biol.

[13] Tatonetti NP, Patrick PY, Daneshjou R, Altman RB (2012). Data-driven prediction of drug effects and interactions. Sci Trans Med.

[14] Kanehisa M, Goto S, Furumichi M, Tanabe M, Hirakawa M (2009). Kegg for representation and analysis of molecular networks involving diseases and drugs. Nucleic Acids Res.

[15] Poluzzi E, Raschi E, Moretti U, De Ponti F (2009). Drug-induced torsades de pointes: data mining of the public version of the fda adverse event reporting system (aers). Pharmacoepidemiol Drug Safety.

[16] Vilar S, Harpaz R, Uriarte E, Santana L, Rabadan R, Friedman C (2012). Drug-drug interaction through molecular structure similarity analysis. J Am Med Inf Assoc.

[17] Liu L, Chen L, Zhang Y. -H., Wei L, Cheng S, Kong X, Zheng M, Huang T, Cai Y-D (2017). Analysis and prediction of drug–drug interaction by minimum redundancy maximum relevance and incremental feature selection. J Biomole Struct Dynamics.

[18] Cheng F, Zhao Z (2014). Machine learning-based prediction of drug–drug interactions by integrating drug phenotypic, therapeutic, chemical, and genomic properties. J Am Med Inf Assoc.

[19] Vilar S, Uriarte E, Santana L, Tatonetti NP, Friedman C (2013). Detection of drug-drug interactions by modeling interaction profile fingerprints. PloS one.

[20] Takeda T, Hao M, Cheng T, Bryant SH, Wang Y (2017). Predicting drug–drug interactions through drug structural similarities and interaction networks incorporating pharmacokinetics and pharmacodynamics knowledge. J Cheminforma.

[21] Vilar S, Uriarte E, Santana L, Friedman C, P Tatonetti N (2014). State of the art and development of a drug-drug interaction large scale predictor based on 3d pharmacophoric similarity. Curr Drug Metabolism.

[22] Herrero-Zazo M, Segura-Bedmar I, Hastings J, Martínez P (2015). Dinto: using owl ontologies and swrl rules to infer drug–drug interactions and their mechanisms. J Chem Inf Modeling.

[23] Li P, Huang C, Fu Y, Wang J, Wu Z, Ru J, Zheng C, Guo Z, Chen X, Zhou W (2015). Large-scale exploration and analysis of drug combinations. Bioinformatics.

[24] Ferdousi R, Safdari R, Omidi Y (2017). Computational prediction of drug-drug interactions based on drugs functional similarities. J Biomed Informa.

[25] Sridhar D, Fakhraei S, Getoor L (2016). A probabilistic approach for collective similarity-based drug–drug interaction prediction. Bioinformatics.

[26] Zhang P, Wang F, Hu J, Sorrentino R (2015). Label propagation prediction of drug-drug interactions based on clinical side effects. Sci Rep.

[27] Cao D-S, Xiao N, Li Y-J, Zeng W-B, Liang Y-Z, Lu A-P, Xu Q-S, Chen A (2015). Integrating multiple evidence sources to predict adverse drug reactions based on a systems pharmacology model. CPT: Pharmacom Syst Pharmacol.

[28] Chen X, Ren B, Chen M, Wang Q, Zhang L, Yan G (2016). Nllss: predicting synergistic drug combinations based on semi-supervised learning. PLoS Comput Biol.

[29] Zhang W, Chen Y, Liu F, Luo F, Tian G, Li X (2017). Predicting potential drug-drug interactions by integrating chemical, biological, phenotypic and network data. BMC bioinformatics.

[30] Matthews EJ, Frid AA (2010). Prediction of drug-related cardiac adverse effects in humans-a: Creation of a database of effects and identification of factors affecting their occurrence. Reg Toxicol Pharmacol.

[31] Zakharov AV, Varlamova EV, Lagunin AA, Dmitriev AV, Muratov EN, Fourches D, Kuz’min VE, Poroikov VV, Tropsha A, Nicklaus MC (2016). Qsar modeling and prediction of drug–drug interactions. Mole Pharma.

[32] Cami A, Manzi S, Arnold A, Reis BY (2013). Pharmacointeraction network models predict unknown drug-drug interactions. PloS one.

[33] Huang H, Zhang P, Qu XA, Sanseau P, Yang L (2014). Systematic prediction of drug combinations based on clinical side-effects. Sci Rep.

[34] Tari L, Anwar S, Liang S, Cai J, Baral C (2010). Discovering drug–drug interactions: a text-mining and reasoning approach based on properties of drug metabolism. Bioinformatics.

[35] Iyer SV, Harpaz R, LePendu P, Bauer-Mehren A, Shah NH (2014). Mining clinical text for signals of adverse drug-drug interactions. J Am Med Informa Assoc.

[36] Banda JM, Callahan A, Winnenburg R, Strasberg HR, Cami A, Reis BY, Vilar S, Hripcsak G, Dumontier M, Shah NH (2016). Feasibility of prioritizing drug–drug-event associations found in electronic health records. Drug Safety.

[37] Chen L, Chu C, Zhang Y-H, Zheng M, Zhu L, Kong X, Huang T (2017). Identification of drug-drug interactions using chemical interactions. Curr Bioinforma.

[38] Wang J, Kribelbauer J, Rabadan R (2016). Network propagation reveals novel features predicting drug response of cancer cell lines. Curr Bioinforma.

[39] Liu H, Luo L, Cheng Z, Sun J, Guan J, Zheng J, Zhou S (2018). Group-sparse modeling drug-kinase networks for predicting combinatorial drug sensitivity in cancer cells. Curr Bioinforma.

[40] Öztürk H, Özgür A, Ozkirimli E (2018). Deepdta: deep drug–target binding affinity prediction. Bioinformatics.

[41] van Laarhoven T, Nabuurs SB, Marchiori E (2011). Gaussian interaction profile kernels for predicting drug–target interaction. Bioinformatics.

[42] Yan C, Wang J, Lan W, Wu F-X, Pan Y (2017). Sdtrls: Predicting drug-target interactions for complex diseases based on chemical substructures. Complexity.

[43] Lan W, Li M, Zhao K, Liu J, Wu F-X, Pan Y, Wang J (2016). Ldap: a web server for lncrna-disease association prediction. Bioinformatics.

[44] Yan C, Wang J, Ni P, Lan W, Wu FX, Pan Y (2019). Dnrlmf-mda:predicting microrna-disease associations based on similarities of micrornas and diseases. IEEE/ACM Trans Comput Biol Bioinforma.

[45] Yan C, Duan G, Wu F, Pan Y, Wang J. Brwmda: Predicting microbe-disease associations based on similarities and bi-random walk on disease and microbe networks. IEEE/ACM Trans Comput Biol Bioinforma. 2019. 10.1109/TCBB.2019.2907626. (to be published). 10.1109/TCBB.2019.2907626 30932846

[46] Yan C, Duan G, Wu F, Pan Y, Wang J. Mchmda: Predicting microbe-disease associations based on similarities and low-rank matrix completion. IEEE/ACM Trans Comput Biol Bioinforma. 2019. 10.1109/TCBB.2019.2926716. (to be published). 10.1109/TCBB.2019.2926716 31295117

[47] Wang B, Mezlini AM, Demir F, Fiume M, Tu Z, Brudno M, Haibe-Kains B, Goldenberg A (2014). Similarity network fusion for aggregating data types on a genomic scale. Nat Methods.

[48] Xia Z, Wu L-Y, Zhou X, Wong ST (2010). Semi-supervised drug-protein interaction prediction from heterogeneous biological spaces. BMC Syst Biol.

[49] Yan C, Wang J, Wu F-X (2018). Dwnn-rls: regularized least squares method for predicting circrna-disease associations. BMC bioinformatics.

[50] Wu Z, Cheng F, Li J, Li W, Liu G, Tang Y (2016). Sdtnbi: an integrated network and chemoinformatics tool for systematic prediction of drug–target interactions and drug repositioning. Brief Bioinforma.

[51] Luo H, Wang J, Li M, Luo J, Peng X, Wu F-X, Pan Y (2016). Drug repositioning based on comprehensive similarity measures and bi-random walk algorithm. Bioinformatics.

[52] Luo H, Li M, Wang S, Liu Q, Li Y, Wang J (2018). Computational drug repositioning using low-rank matrix approximation and randomized algorithms. Bioinformatics.

[53] Luo H, Wang J, Li M, Luo J, Ni P, Zhao K, Wu FX, Pan Y. Computational drug repositioning with random walk on a heterogeneous network. IEEE/ACM Trans Comput Biol Bioinforma. 2018. 10.1109/TCBB.2018.2832078. (to be published). 10.1109/TCBB.2018.2832078 29994051

[54] Lu C, Yang M, Luo F, Wu F-X, Li M, Pan Y, Li Y, Wang J (2018). Prediction of lncrna-disease associations based on inductive matrix completion. Bioinformatics.

[55] Lan W, Wang J, Li M, Liu J, Wu F-X, Pan Y (2018). Predicting microrna-disease associations based on improved microrna and disease similarities. IEEE/ACM Trans Comput Biol Bioinforma.

[56] Wishart DS, Feunang YD, Guo AC, Lo EJ, Marcu A, Grant JR, Sajed T, Johnson D, Li C, Sayeeda Z (2017). Drugbank 5.0: a major update to the drugbank database for 2018. Nucleic Acids Res.

[57] Bhattacharya A, Bandichhor R. Green technologies in the generic pharmaceutical industry. Green Chem Pharma Ind. 2010:304–6.

[58] Mauss S, Klinker H (2013). Drug-drug interactions in the treatment of hcv among people who inject drugs. Clin Inf diseases.

[59] Welage LS, Berardi RR (2000). Evaluation of omeprazole, lansoprazole, pantoprazole, and rabeprazole in the treatment of acid-related diseases. J Am Pharma Assoc 1996.

[60] Vítovec J, Špinar J (2000). First-dose hypotension after angiotensin-converting enzyme (ace) inhibitors in chronic heart failure: a comparison of enalapril and perindopril. Eur J Heart Fail.

[61] Burmeister WE, Reynolds RD, Lee RJ (1981). Limitation of myocardial infarct size by atenolol, nadolol and propranolol in dogs. Eur J Pharmacol.

[62] Reeves RA, From GL, Paul W, Leenen FH (1985). Nadolol, propranolol, and thyroid hormones: Evidence for a membrane-stabilizing action of propranolol. Clin Pharmacol Therap.

[63] UCHIDA E, OGUCHI K, HISAOKA M, KOBAYASHI S, KAI K, YASUHARA H (1988). Effects of ranitidine, metoclopromide, and anisotropine methylbromide on the availability of cefpodoxime proxetil (cs-807) in japanese healthy subjects. Rinsho yakuri/Japan J Clin Pharmacol Therap.

[64] El-Beqqali A, Kussak A, Blomberg L, Abdel-Rehim M (2007). Microextraction in packed syringe/liquid chromatography/electrospray tandem mass spectrometry for quantification of acebutolol and metoprolol in human plasma and urine samples. J Liquid Chromatogr Rel Technol.

[65] Florentin M, Elisaf MS (2012). Simvastatin interactions with other drugs. Exp Opin Drug Safety.

[66] Treyger G, Silver SA, Sakharova AA (2015). Pheochromocytoma diagnosis after an abnormal stress test: Case report and review of the literature. J.

[67] Yang M, Luo H, Li Y, Wang J (2019). Drug repositioning based on bounded nuclear norm regularization. Bioinformatics.

[68] Liu J, Pan Y, Li M, Chen Z, Tang L, Lu C, Wang J (2018). Applications of deep learning to mri images: A survey. Big Data Mining Anal.

[69] Liu L, Yu Y, Fei Z, Li M, Wu F-X, Li H-D, Pan Y, Wang J (2018). An interpretable boosting model to predict side effects of analgesics for osteoarthritis. BMC Syst Biol.

[70] Chang C-C, Lin C-J (2011). Libsvm: a library for support vector machines. ACM Trans Intell Syst Technol (TIST).

[71] Lin C, Chen W, Qiu C, Wu Y, Krishnan S, Zou Q (2014). Libd3c: ensemble classifiers with a clustering and dynamic selection strategy. Neurocomputing.

[72] Wang H, Wang J, Zhou L (2018). A survival ensemble of extreme learning machine. Appl Intell.

[73] Luo H, Wang J, Yan C, Li M, Fangxiang W, Yi P. A novel drug repositioning approach based on collaborative metric learning. IEEE/ACM Trans Comput Biol Bioinforma. 2019. 10.1109/TCBB.2019.2926453. (to be published). 10.1109/TCBB.2019.2926453 31283509

